# Familiar cylindromatosis in a Colombian family caused by a mutation in CYLD

**DOI:** 10.3332/ecancer.2024.1768

**Published:** 2024-09-13

**Authors:** Lisa Ximena Rodríguez Rojas, Diana Vasquez-Forero, Juan José Albán, Liliana Doza, Sandra Murillo, Jorge Andrés Olave-Rodriguez, José Nastasi

**Affiliations:** 1 Department of Human Genetics, Fundación Valle del Lili, Cali 760026, Colombia; 2 Faculty of Health Sciences, ICESI University, Cali 760031, Colombia; 3 Center of Clinical Research, Fundación Valle del Lili, Cali 760026, Colombia

**Keywords:** CYLD, cylindroma, CYLD cutaneous syndrome

## Abstract

**Introduction:**

The CYLD cutaneous syndrome is characterised by the appearance of multiple skin tumours, including cylindromas, spiradenomas, trichoepitheliomas and basal cell adenomas of the salivary gland and less frequently pulmonary cylindromas. The lesions appear in the second decade of life, typically present as single lesions, located mainly on the face and head and progressively increase in number, potentially affecting the torso, groin and axillae. Although lesions can affect both men and women, a higher frequency of affected women has been described.

**Case presentation:**

CYLD cutaneous syndrome is caused by pathogenic variants in the CYLD gene, following an autosomal dominant inheritance pattern. We present the first Colombian case of a family affected by CYLD cutaneous syndrome, spanning three generations and characterised by early onset of skin lesions. This syndrome was molecularly confirmed by next-generation sequencing (NGS), reveling a heterozygous frameshift variant in the CYLD gene, specifically the type NM_015247.2 c.2291_2295delAACTA p.Lys764Ilefs*2, which was subsequently confirmed by Sanger sequencing.

**Conclusion:**

Understanding the complex interplay of genetic, epigenetic and environmental factors in the malignant transformation of cylindroma to squamous eccrine ductal carcinoma is crucial for developing targeted therapies and improving patient outcomes.

**Key messages:**

The CYLD cutaneous syndrome in a Colombian family.

## Introduction

The CYLD cutaneous syndrome is characterised by the presence of benign cutaneous tumours such as cylindromas, spiradenomas and trichoepitheliomas [1]. It is less frequently associated with basal cell adenomas of the salivary gland and pulmonary cylindromas [1, 3]. Previously described as separate entities, Brooke-Spiegler syndrome, familial cylindromatosis and familial multiple trichoepithelioma [1], it is currently established that they are part of the same clinical spectrum, given their variable intra- and inter-familial expressivity [1]. A hormonal etiology has been suggested due to greater severity in women, although the mechanism has not yet been elucidated [1].

This syndrome is caused by heterozygous pathogenic germline variants in the CYLD gene [2], which is located on chromosome 16q12.1. The CYLD gene comprises 20 exons and encodes a 956-amino acid protein with deubiquitinase enzymatic activity. It possesses two functional domains: three cytoskeleton-associated glycin**e**-rich domains (CAP-GLY) and a catalytic domain in the C-terminal region known as the ubiquitin-specific protease domain [3], responsible for deubiquitinase activity and where most described mutations are found [4]. The primary function is the posttranslational modification of proteins by removing ubiquitin attached to Lys63. It exerts negative regulation upon multiple cellular survival pathways, including the nuclear factor-kB signaling pathway [5] and the MAP kinase pathway, by removing ubiquitin chains from molecules such as NEMO, TRAF2, TRAF6 and RIPK1 [4]. CYLD has been classified as a tumour suppressor gene and has a crucial role in cancer pathogenesis. This gene has been implicated in melanoma, colorectal carcinoma, hepatocellular carcinoma and leukemia [2].

Cylindromas, spiradenomas and trichoepitheliomas are classified as benign tumours with potential for malignant transformation into basal cell carcinoma, which is the most common outcome. In some cases, they may progress to invasive adenocarcinoma, sarcomatous carcinoma [1, 3] and in rare cases, squamous cell carcinoma [3]. Cylindroma, traditionally viewed as a benign adnexal tumour with a predilection for the scalp and face, is characterised by its histological appearance, often described as ‘jigsaw puzzle-like’ islands of basaloid cells in the dermis. These tumours are generally considered benign, but there is a rare potential for malignant transformation. The transition from cylindroma to malignant squamous eccrine ductal carcinoma is a rare and actively researched area. This process involves understanding the molecular, genetic and environmental factors that drive malignancy in adnexal tumours. The CYLD gene, a tumour suppressor that negatively regulates the NF-κB pathway and other cell proliferation and survival pathways, plays a crucial role. Loss of CYLD function can result in uncontrolled cellular proliferation, which is a hallmark of cancer. During malignant transformation, additional genetic alterations may occur, facilitating the progression from benign cylindroma to squamous eccrine ductal carcinoma, including mutations in other tumour suppressor genes or oncogenes affecting cell cycle, apoptosis and DNA repair mechanisms. Epigenetic changes such as DNA methylation and histone modification also play a vital role, potentially silencing tumour suppressor genes or activating oncogenes. In the transformation to squamous eccrine ductal carcinoma, microenvironmental changes could promote invasion, angiogenesis and immune surveillance evasion. Aberrant activation or suppression of critical signaling pathways such as Wnt, Notch and Hedgehog, which regulate skin development and homeostasis, could be key in malignant transformation, affecting cell fate, proliferation and differentiation. Specifically, dysregulation in these pathways could drive the transition from a benign adnexal tumour to a more aggressive and invasive carcinoma, with changes in tumour cells that allow them to avoid immune detection and destruction being critical. These lesions typically present ulceration, local bleeding, rapid tumour growth and intense pain and have a high risk of metastasis to lymph nodes [2], bone, lung and liver [1, 3]. Somatic studies have revealed homozygous mutations of CYLD in these tumours, suggesting a ‘two-hit’ mechanism where an initial pathogenic germline mutation is followed by the acquisition of a second pathogenic somatic mutation, leading to the development of cutaneous adnexal tumours and in less frequently, basal cell adenomas of the salivary glands can transform into adenocarcinomas [3]. Some patients have mosaic CYLD mutations, which result in the development of unilateral tumours clustered in specific locations. These individuals typically do not have a family history of the condition and diagnosis requires biopsy studies of the lesion [3].

## Case description

**Figure 1.**
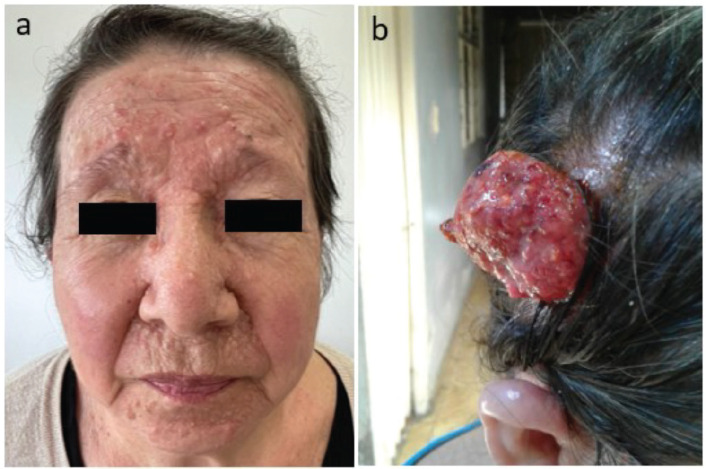
(a): Trichoepitheliomas in the frontal region of the patient´s face. (b): Malignant adnexal eccrine tumour consistent with spiradenocarcinoma in the right temporal region of the patient’s scalp.

We present a 65-year-old woman, with non-consanguineous parents, with a clinical history that began at 15 years of age with the appearance of pearl-like nodular lesions approximately 0.5 cm in size, primarily located on the nose, nasolabial folds and upper lips. These lesions progressively increased in number. Surgical resection was performed at the age of 18, but the lesions recurred. In 2021, surgical resection of some lesions was performed and histopathological examination revealed trichoepitheliomas (Figure 1a). Subsequently, at the age of 63, a tumour lesion of approximately 10 × 5 cm was found on the scalp (Figure 1b) and was excised.

**Figure 2.**
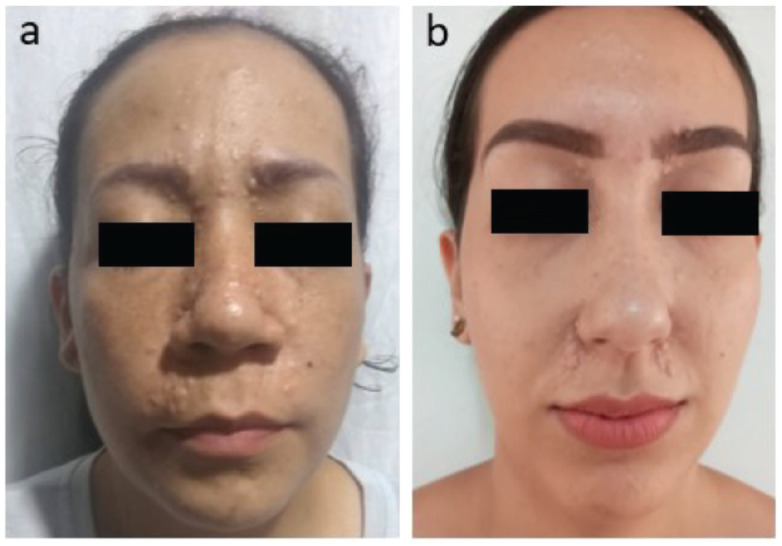
(a): Trichoepitheliomas around the nose, mouth and in the frontal region of the face in patient’s daughter. (b): Trichoepitheliomas around the nose, mouth and in frontal region of the face in patient’s granddaughter.

The histological study reveals an adnexal tumour with basaloid epithelial differentiation formed by a lobule of small cells with hyperchromatic nuclei of scant cytoplasm, with differentiation to small groups of squamous cells with abundant mitoses and areas of necrosis. The immunohistochemical analysis demonstrates tumour cells positive for cytokeratin AE1/AE3 and CEAp, while the foci of squamous differentiation show positivity for EMA and P63. Biphasic expression of CAM 5.2 is observed in the eccrine component and cytokeratin 14 is accentuated in the squamous component. These findings are compatible with a malignant adnexal eccrine tumour consistent with spiradenocarcinoma. Up to now, the patient has not received radiation therapy or chemotherapy. A brain magnetic resonance imaging was performed with no evidence of intracranial lesions. The patient has three children: the eldest daughter is 42 years old and does not have any lesions. The 38-year-old daughter has similar lesions on her face (Figure 2a), who has two children: a 14-year-old son without lesions and a 21-year-old daughter with facial lesions (Figure 2b).

**Figure 3.**
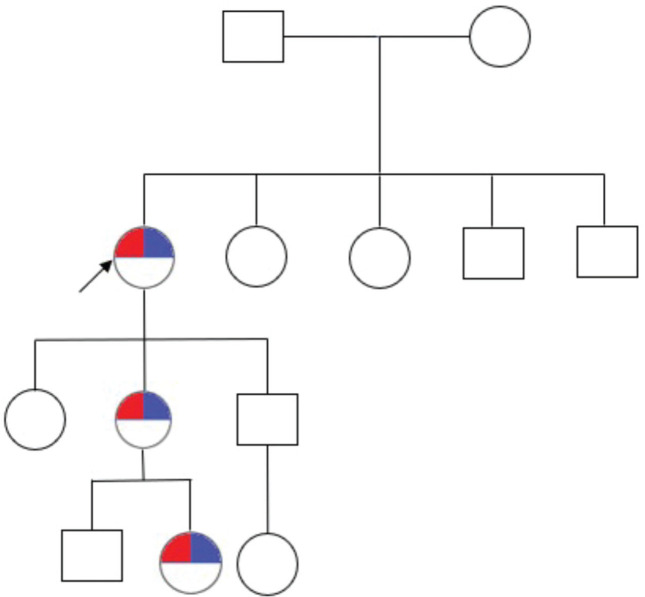
Family pedigree. The arrowhead points to the patient. Furthermore, the red marking refers to the phenotype of CYLD cutaneous syndrome and the blue marking refers to the genotype of CYLD cutaneous syndrome (CYLD (NM_015247.2) c.2291_2295AACTA p.Lys764Ilefs*2).

The 33-year-old son does not have any lesions and he has an 8-year-old daughter without lesions. The family pedigree consists of three generations and includes seven individuals, with three of them being affected across the three generations (Figure 3).

Due to the clinical course and family history of the patient, a molecular study using a multigene panel for cancer predisposition genes including the CYLD gene, was performed. This genetic study was performed on the patient and her affected family members. A pathogenic variant in the heterozygous state of the CYLD gene was identified in the patient and her affected family member, mutation CYLD (NM_015247.2) c.2291_2295delAACTA p.

**Figure 4.**
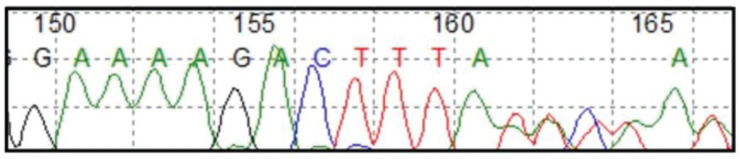
CYLD c.2291_2295AACTA p.Lys764Ilefs*2 by Sanger sequencing.

Lys764Ilefs*2, this mutation results in the exchange of a lysine for an isoleucine at position 764 of the protein which causes a frameshift two amino acids downstream. This mutation has been reported in ClinVar as pathogenic (VCV000267248.1) [6]. Molecular confirmation was obtained through Sanger sequencing (Figure 4), leading to the diagnosis of CYLD cutaneous syndrome.

## Discussion

Our patient was diagnosed with CYLD cutaneous syndrome, with a molecular finding of a variant in the CYLD gene. This variant shows a pathogenic heterozygous deletion of five base pairs in exon 7 of the gene, resulting in a frameshift mutation leading to a premature stop codon in the protein. The presence of this variant, in association with the characteristic phenotype, confirms the diagnosis of CYLD cutaneous syndrome.

More than 100 different mutations have been reported in the literature, [7] with the majority being frameshift variants followed by nonsense variants. These mutations are located in exons 16, 17 and 20 [8], which encode the catalytic domain of the protein. They result in truncated proteins without deubiquitinase activity, leading to haploinsufficiency of the CYLD gene [1, 4]. Some reports suggest that missense mutations have a milder phenotype [4], but so far, no genotype-phenotype correlation has been established [8]. Additionally, mutations do not seem to predict severity [3] as there is significant intra- and inter-familial variability in both phenotypic presentation and tumour count [3]. At the moment, there is no specific treatment available to achieve control of the lesions. Various approaches have been proposed, including dermabrasion, Nd:YAG laser, trichloroacetic acid, thermocauterization and intralesional injection of triamcinolone acetonide. However, these methods have not been recommended due to the high recurrence rate and the inability to eradicate the lesions [9]. Some authors suggest early surgical resection with flap reconstruction as a means to prevent recurrences [9], but this approach has resulted in significant aesthetic complications for patients. Understanding the complex interplay of genetic, epigenetic and environmental factors in the malignant transformation of cylindroma to squamous eccrine ductal carcinoma is crucial for developing targeted therapies and improving patient outcomes. Further research in this area is essential to unravel the precise mechanisms involved and to identify potential biomarkers for early detection and treatment.

## Conflicts of interest

The authors have no conflicts of interest to declare.

## Funding

This study was not supported by any sponsor or funder.

## Statement of ethics

This study protocol was reviewed and approved by Comité de Ética en Investigación Biomédica of the Fundación Valle del Lili, approval number 591. Written informed consent was obtained from the patient for publication of this case report and any accompanying images.

## Author contributions

LXR, DMVF and JJA data curation, formal analysis, investigation, methodology, validation, writing – original draft and writing – review and editing JANC and LD conceptualization, data curation, formal analysis, investigation, methodology, validation, writing – original draft and writing – review and editing and SEM and JAO edited the manuscript.

## Data availability statement

All data generated or analysed during this study are included in this article. Further enquiries can be directed to the corresponding author.
